# Epigenetic pharmacology in aging: from mechanisms to therapies for age-related disorders

**DOI:** 10.3389/fphar.2025.1699296

**Published:** 2025-10-30

**Authors:** Haikuan Yu, Taojin Feng, Chengcheng Zhang, Zhouguang Jiao, Wenkai Fan, Rongxian Jiang, Dewen Kong, Fubing Li

**Affiliations:** ^1^ Department of Orthopedics, The 927th Hospital of Joint Logistics Support Force of People’s Liberation Army of China, Puer, China; ^2^ Department of Orthopedics, The Fourth Medical Center of Chinese PLA General Hospital, Beijing, China; ^3^ Department of Orthopedics, The 920th Hospital of Joint Logistics Support Force of People’s Liberation Army of China, Kunming, Yunnan, China; ^4^ NHC Key Laboratory of Human Disease Comparative Medicine, Institute of Laboratory Animal Sciences, Peking Union Medicine College, Chinese Academy of Medical Sciences, Beijing, China; ^5^ School of Teacher Education, Pu’er University, Puer, China

**Keywords:** aging, epigenetics, age-related diseases, pharmacological epigenetics, DNA methylation, histone modification, non-coding RNA, epigenetic modifiers

## Abstract

Aging is a multidimensional process regulated by the interplay of genetic and environmental factors, with epigenetic alterations serving as a central regulatory hub. Aberrant DNA methylation patterns, dysregulation of histone-modifying enzymes (e.g., SIRT1, EZH2), and non-coding RNA-mediated mechanisms collectively remodel gene expression networks, impacting critical pathways such as cellular senescence and mitochondrial homeostasis. This establishes an “environment-epigenome-disease” causal axis, closely associated with pathologies including β-amyloid deposition in Alzheimer’s disease, atherosclerosis, immunosenescence, osteoporosis, sarcopenia, and tumorigenesis. Capitalizing on the reversible nature of epigenetic modifications, pharmacological epigenetics has emerged as a cutting-edge field for intervening in aging and age-related diseases. Targeting key epigenetic modifiers such as DNA methyltransferases and histone deacetylases enables the modulation of disease-associated epigenetic states, providing a promising avenue for therapeutic intervention in aging and age-related diseases. This review synthesizes the molecular mechanisms of epigenetic regulation in aging, their role in age-related diseases, and advances in pharmacological epigenetics—from basic research to clinical translation. It further situates key challenges such as target specificity, long-term safety, and tissue-specific delivery within a translational framework, aiming to inform strategies for the diagnosis and intervention of age-related conditions.

## 1 Introduction

With the profound transformation of the global population structure, aging and age-related diseases have emerged as significant challenges in the field of public health in the 21st century. According to a report by the World Health Organization (WHO), the global population aged 60 years and older has increased from 600 million in 2000 to 1 billion in 2023, and is projected to reach 2.1 billion by 2050, accounting for 22% of the total population (WHO, 2025). This acceleration of population aging is accompanied by a notable rise in the incidence of age-related diseases, including cardiovascular diseases (CVDs), neurodegenerative disorders (such as Alzheimer’s disease (AD) and Parkinson’s disease (PD)), malignant tumors, type 2 diabetes mellitus (T2DM), immune dysfunction, osteoporosis, and disorders related to mitochondrial dysfunction (Caruso et al., 2021). These diseases not only increase disability rates among the elderly and shorten healthy life expectancy but also lead to excessive consumption of medical resources. Chronic diseases and mental health disorders consume the vast majority of America’s healthcare resources, representing 90% of the nation’s $4.9 trillion annual medical spending (CDC, 2025). From a socioeconomic perspective, the surge in long-term care demand is projected to result in a global shortage of 13 million caregivers by 2050. Additionally, the decline in working-age populations—where those aged 65 and older already exceed 28% in some developed countries—exerts dual pressures on social security systems and sustainable economic development (Schwarz, 2021; madisontrust, 2025). Therefore, systematically analyzing the biological mechanisms of aging and developing targeted intervention strategies have become core scientific issues in addressing global health crises.

Aging is a multi-dimensional biological process characterized by declining cellular functions, disrupted tissue homeostasis, and organ dysfunction. This process has been summarized into 12 hallmarks: epigenetic alterations, genomic instability, telomere shortening, loss of proteostasis, autophagic dysfunction, dysregulated nutrient sensing, mitochondrial dysfunction, cellular senescence, stem cell exhaustion, altered intercellular communication, chronic inflammation, microbiota dysbiosis (López-Otín et al., 2023). In 2025, Carlos López-Otin expanded the characteristics of aging to 14 hallmarks, adding extracellular matrix changes and psychosocial isolation (Kroemer et al., 2025). Among these hallmarks, epigenetic changes are dynamically regulated by the interaction between genetic factors and environmental exposures. As a key bridge linking genotype to phenotype, epigenetics plays a central role in shaping the trajectory of aging. This regulatory effect of epigenetics is primarily achieved through the modulation of gene expression. Specifically, gene expression regulation in this context is mediated by three core epigenetic mechanisms: DNA methylation, post-translational modifications of histones (e.g., acetylation and methylation), and non-coding RNAs (ncRNAs)—including microRNAs (miRNAs) and long non-coding RNAs (lncRNAs) (Kroemer et al., 2025). Altered DNA methylation patterns are commonly observed in senescent cells; notable examples include hypermethylation of CpG islands and global hypomethylation of the genome. These age-related DNA methylation alterations can activate the senescence-associated secretory phenotype (SASP) by remodeling the transcriptome (Dasgupta et al., 2024). Furthermore, the dysregulation of histone-modifying enzymes (such as Sirtuin 1 (SIRT1) and enhancer of zeste homolog 2 (EZH2)) directly participates in core aging pathways, including the maintenance of telomere function and the regulation of mitochondrial homeostasis (la Torre et al., 2023; Dasgupta et al., 2024). Notably, environmental factors (such as dietary restriction, chronic inflammation, and pollutant exposure) can induce transgenerational effects or age-related phenotypic changes through epigenetic modifications, thereby forming a causal chain of “environment-epigenome-disease” (Tzeng and Lee, 2024; Nicholls et al., 2025). In disease association studies, epigenetic dysregulation has been confirmed to be closely linked to β-amyloid deposition in AD, phenotypic switching of vascular smooth muscle cells in atherosclerosis, and genomic instability in tumors. These findings reveal not only the epigenetic plasticity associated with aging but also offer new directions for the screening of early diagnostic markers and the identification of potential intervention targets (Li et al., 2019; Li J. Z. et al., 2024; Vasile and Rouach, 2023; Janic et al., 2025; Lambert and Jørgensen, 2025).

Given the reversible nature of epigenetic regulation, pharmacological epigenetics seeks to intervene in disease-related gene expression networks by targeting epigenetic modifying enzymes or regulatory elements, emerging as a frontier in the treatment of aging and age-related diseases (Hernández-Saavedra et al., 2019). This discipline integrates medicinal chemistry, molecular biology, and epigenomics to systematically investigate the remodeling effects of DNA methyltransferase (DNMT) inhibitors (such as decitabine), histone deacetylase (HDAC) inhibitors (such as vorinostat), and RNA interference therapies on epigenetic states (Li J. Z. et al., 2024). At the basic research level, the combination of high-resolution epigenomic sequencing technologies (such as assay for transposase-accessible chromatin using sequencing (ATAC-seq) and chromatin immunoprecipitation sequencing (ChIP-seq) with organoid models has facilitated precise analyses of chromatin dynamic remodeling under drug action (Li J. Z. et al., 2024; Niu et al., 2024; Janic et al., 2025). In the realm of clinical translation, epigenetic drugs have demonstrated remarkable efficacy in the treatment of hematological tumors (Bates, 2020). DNMT inhibitors are effective in treating MDS, as evidenced by their ability to achieve clinical responses through DNA hypomethylation, with oral decitabine plus cedazuridine showing efficacy comparable to intravenous decitabine (Savona et al., 2019). However, their clinical applicability outside oncology (e.g., in neurodegenerative or metabolic diseases) remains highly limited. Furthermore, ongoing clinical trials of HDAC inhibitors in combination with immunotherapy for solid tumors, such as those registered under NCT03298905 (gov.uk, 2025), are revealing synergistic potential. Notably, studies focused on reprogramming aging-related epigenetic clocks, including DNA methylation age, have shown partial tissue rejuvenation in mouse models, providing proof-of-concept for the possibility of delaying the aging process (Browder et al., 2022). However, challenges remain, including issues related to target specificity, long-term safety, and cross-tissue delivery efficiency of epigenetic drugs, which necessitate continuous optimization through multidisciplinary research.

**FIGURE 1:**
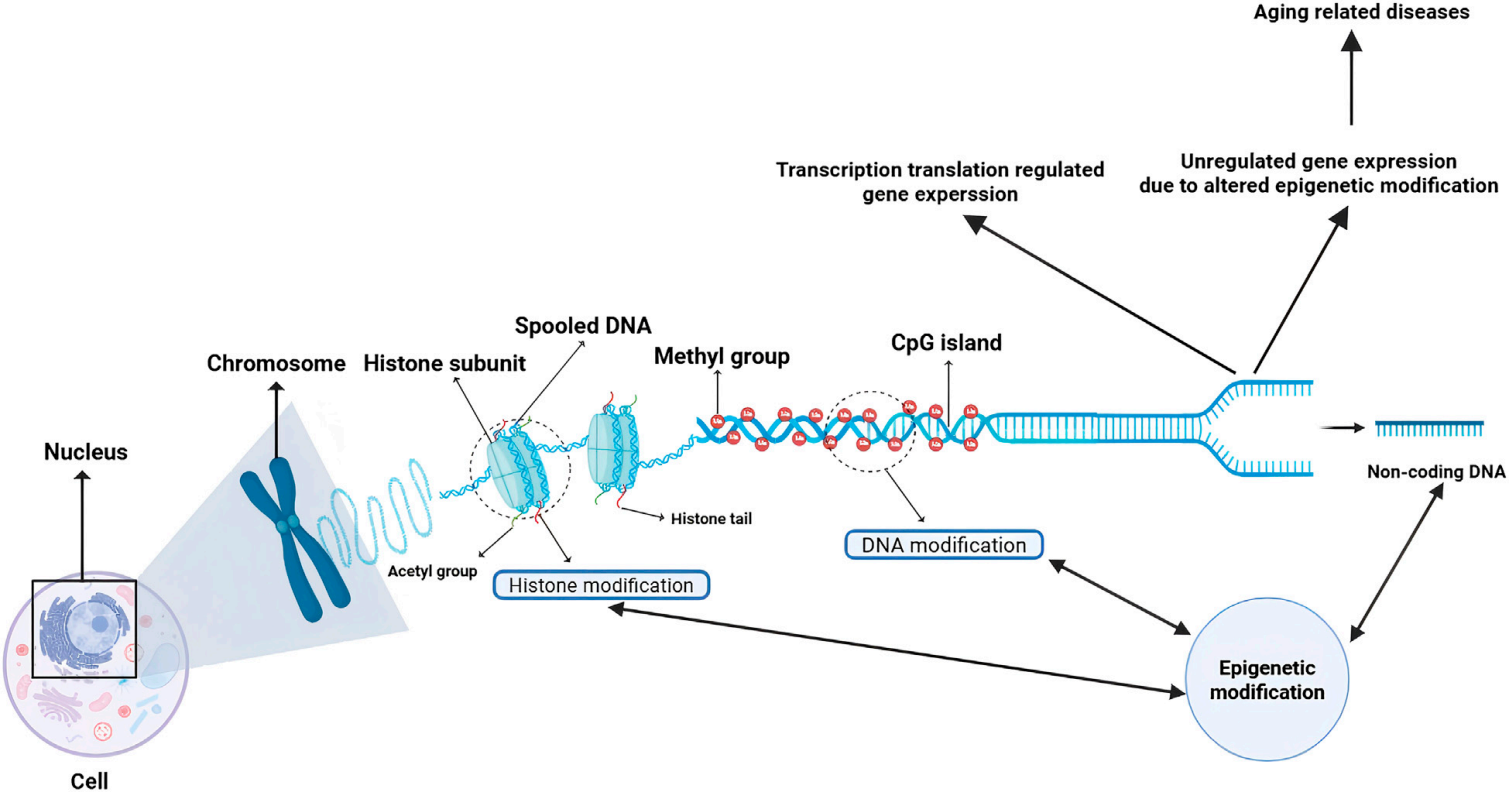
Schematic of epigenetic gene regulation and its role in aging-related diseases. This diagram illustrates the core mechanisms of epigenetic regulation within the nucleus. DNA is spooled around histone subunits to form chromosomes. Gene expression is regulated through: Histone modifications (e.g., acetylation/methylation) on histone tails, governed by enzymes such as HATs and HDACs, which modulate chromatin accessibility. DNA methylation at CpG islands, catalyzed by DNMTs, which typically represses transcription. Actions of non-coding RNAs derived from non-coding DNA, which provide an additional layer of post-transcriptional control. The dysregulation of these processes—altering the delicate balance of transcription and translation—disrupts normal gene expression patterns, ultimately driving the pathogenesis of a spectrum of age-related diseases, such as cancer, neurodegenerative disorders, cardiovascular diseases, immune dysfunction, and metabolic syndromes (Wang et al., 2022). (Created with BioRender.com). Abbreviations: HATs, Histone Acetyltransferases; HDACs, Histone Deacetylases; DNMTs, DNA Methyltransferases.

This review will systematically elaborate the core content of epigenetics, analyze the molecular mechanism of epigenetics, and analyze how DNA methylation, histone modification and non-coding RNA regulate gene expression. Then, the close relationship between epigenetic disorders and age-related diseases such as AD and tumor is discussed, and its key role in the occurrence and development of diseases is revealed (Figure 1). This paper expounds the frontier progress of pharmacology epigenetics, covering basic research breakthroughs and clinical transformation results, and discusses the existing challenges and future optimization direction, so as to comprehensively show the research trend and development potential in this field.

## 2 Epigenetic advances and therapeutic applications in age-related diseases

### 2.1 DNA methylation and the epigenetic clock

In aging research, the significance of epigenetics is increasingly acknowledged. DNA methylation, a core mechanism of epigenetic regulation, plays a crucial role in the aging process. This process involves the addition of a methyl group to the C-5 position of cytosine (C), typically within CpG dinucleotides, and is catalyzed by DNA methyltransferases (DNMTs). Such modifications can influence gene expression patterns, thereby regulating cellular functions and fates. As individuals age, DNA methylation patterns undergo substantial alterations, which are not only closely associated with age-related physiological decline but also pave the way for epigenetic-based interventions aimed at combating aging (Crane et al., 2019; Schaffer et al., 2023).

The connection between DNA methylation and aging was first established in yeast models. Research has demonstrated that during the aging process in yeast, changes in histone modifications and DNA methylation result in altered gene expression patterns, which in turn accelerate cellular senescence (Perez et al., 2024). In mammals, this relationship is more intricate. The ‘epigenetic clock’ developed by Horvath et al. accurately predicts an individual’s biological age by analyzing the methylation status of specific CpG sites (Tharakan et al., 2020). This clock demonstrates high consistency across various tissues and can also reflect an individual’s health status and disease risk, thereby serving as a powerful tool in aging research (Zhou et al., 2023). The mechanisms through which DNA methylation influences aging operate at multiple levels. During the aging process, global DNA methylation levels generally decrease, while specific gene promoter regions may become hypermethylated, resulting in gene silencing. For instance, DNA methylation mediated by the Polycomb repressive complex 2 (PRC2) plays a significant role in stem cell aging. PRC2 enhances DNA methylation by catalyzing the trimethylation of histone H3 at lysine 27 (H3K27me3), thereby regulating gene expression (Lan et al., 2019). Research indicates that the methylation level of PRC2 target genes increases with age, a change that is closely associated with the decline in stem cell function (De Cauwer et al., 2022). Furthermore, the methylation status of CCCTC-binding factor (CTCF) binding sites changes with age. CTCF influences the stability of DNA methylation by regulating its “hemimethylated” state, which ultimately leads to alterations in gene expression patterns (Gyenis et al., 2023).

The connection between DNA methylation and aging is evident not only at the cellular level but is also closely linked to various age-related diseases. For instance, in patients with progeria, mutations in DNA repair genes lead to significant alterations in DNA methylation patterns, thereby accelerating the aging process (Yang et al., 2023). In mouse models exhibiting DNA repair deficiencies, the DNA methylation age is markedly increased, highlighting a strong association between DNA damage and epigenetic regulation (Peng et al., 2015). Furthermore, alterations in DNA methylation are closely associated with the development and progression of age-related diseases, including inflammation, metabolic disorders, and cancer (Davalos and Esteller, 2023).

The epigenetic clock constructed based on DNA methylation represents a novel tool for aging research and offers potential targets for anti-aging interventions. Techniques for cellular reprogramming can reset DNA methylation patterns, effectively reversing cellular aging phenotypes (Hananya et al., 2024). Additionally, anti-aging strategies such as dietary restriction and pharmacological interventions have been demonstrated to delay the progression of the DNA methylation clock (Yehuda and Lehrner, 2018; Li C. Z. et al., 2024). This indicates that modulating DNA methylation patterns may hold promise for delaying aging and mitigating age-related diseases. However, despite the encouraging prospects of DNA methylation in aging research, numerous challenges persist. The underlying mechanisms driving changes in DNA methylation remain incompletely understood, and it is still uncertain whether these changes are a cause or merely a consequence of aging (Sen et al., 2016). Moreover, DNA methylation patterns exhibit significant variability across different tissues and cell types, making it crucial to integrate these variations into a unified model of aging, which represents an important direction for future research (Kelsey, 2020).

In summary, DNA methylation, as a fundamental mechanism of epigenetic regulation, plays a critical role in the aging process. The DNA methylation-based epigenetic clock offers novel tools and potential targets for aging research. Future studies must further elucidate the driving mechanisms underlying changes in DNA methylation and investigate their potential applications in anti-aging interventions.

### 2.2 Dynamic imbalance of histone modifications

Histones, which are basic DNA-binding proteins rich in lysine and arginine, form the core scaffold around which DNA is wrapped to assemble into chromatin complexes. The dynamic balance of their modification states is a crucial aspect of epigenetic regulation during the aging process. By altering chromatin compaction, histone modifications directly influence transcriptional efficiency and various cellular physiological processes, thereby playing a central role in cellular senescence and the decline of organ function (Sen et al., 2016). Among these regulatory mechanisms, the imbalance of acetylation—primarily regulated by the HDACs/SIRTs families—and methylation, particularly at typical sites such as H3K27me3 and histone H3 lysine 4 trimethylation (H3K4me3), is of utmost significance and is closely associated with lifespan regulation and age-related functional disorders.

From the perspective of specific imbalance features, abnormalities in histone modifications during aging directly induce disruptions in genomic and cellular homeostasis. For example, H3K4me3 exhibits abnormal accumulation, particularly under energy stress conditions. This dysregulation can lead to aberrant transcriptional elongation, subsequently promoting R-loop formation and genomic instability (Sun et al., 2023); Additionally, histone acetylation levels generally decrease, notably in ribosomal DNA (rDNA) regions. The reduced activity of the histone deacetylase Sir2 directly leads to decreased rDNA stability, resulting in excessive ribosomal RNA (rRNA) production and a collapse of proteostasis. This worsening of chromatin stability and its association with proteostatic challenges become more pronounced with age (Paxman et al., 2022); Furthermore, aberrant methylation of histone H4 lysine 20 (H4K20me) disrupts the balance of BRCA1 and 53BP1 at stalled replication forks during DNA replication and repair, leading to nascent DNA degradation and genomic instability. This phenomenon is particularly evident during aging and can directly accelerate cellular senescence and organ functional decline (Kawarazaki and Fujita, 2021; Xu et al., 2021).

From the perspective of regulatory networks, histone modifications construct multi-dimensional pathways that regulate aging through precise coordination with chromatin accessibility and transcriptional silencing. Histone demethylases target core components of longevity-critical pathways, such as the insulin/insulin-like growth factor-1 (IGF-1) signaling pathway, thereby directly influencing the aging process. SIRT family proteins, which possess both deacetylase and ADP-ribosyltransferase activities, are crucial for maintaining healthy aging. For instance, the overexpression of SIRT1 in transgenic mice does not extend lifespan but effectively improves age-related genomic stability and metabolic efficiency (Sen et al., 2016); Additionally, the overexpression of mitochondria-localized SIRT3 can restore the regenerative capacity of aged hematopoietic stem cells and mediates the beneficial effects of dietary restriction on longevity. SIRT6 demonstrates “bidirectional regulation” characteristics; its gene ablation accelerates aging in mice, while its overexpression significantly extends lifespan. This mechanism is attributed to SIRT6’s role as a multifunctional protein that tightly couples chromatin dynamics with metabolic regulation and DNA repair processes. Conversely, SIRT7 deficiency results in decreased overall genomic stability and metabolic dysfunction, ultimately leading to a progeroid phenotype. Furthermore, genetic inactivation of the histone acetyltransferase KAT7 in human stem cells reduces histone H3 lysine 14 acetylation (H3K14ac) levels, significantly alleviating cellular senescence markers. Intravenous injection of lentiviral vectors encoding Cas9/sg-Kat7 not only ameliorates hepatocyte senescence and liver aging but also extends the lifespan of both normal and progeroid mice. Histone acetyltransferase inhibitors can effectively improve the phenotype of progeroid mice and extend their survival, while histone deacetylase activators, by upregulating SIRT1 activity, partially promote organismal longevity (Sen et al., 2016; Kawarazaki and Fujita, 2021).

The dynamic imbalance of histone modifications is closely linked to the development of age-related diseases. In neurodegenerative disorders, aberrant methylation of histones H3K9 and H3K27 results in dysregulated gene silencing and subsequent neuronal degeneration (Yao et al., 2016); In cancers, such as melanoma, the dysregulation of histone modifications significantly accelerates tumor progression and metastasis by reshaping gene expression programs (Yao et al., 2016). Consequently, interventional strategies targeting histone modifications, including HDAC inhibitors and histone methyltransferase (HMT) inhibitors, have emerged as critical research avenues for anti-aging therapies and the treatment of age-related diseases (Sen et al., 2016; Joo et al., 2025).

The application of single-cell imaging and microfluidic technologies offers novel insights into the dynamic imbalance of histone modifications. Research has demonstrated that the heterogeneity of histone modifications significantly increases in senescent cells, which is closely associated with various pathways of cellular senescence, such as rDNA instability and mitochondrial dysfunction (Paxman et al., 2022); Furthermore, advancements in protein engineering and chromatin reconstitution techniques, including the design of chromatin with specific modification patterns, facilitate molecular-level analyses of the specific effects of histone modifications on aging. This lays a theoretical foundation for the development of interventional strategies (Hananya et al., 2024). Although the critical role of histone modifiers in aging regulation is increasingly acknowledged, their potential as therapeutic targets for age-related cognitive decline requires systematic exploration. A key scientific question remains: do such interventions ultimately regulate aging and longevity solely through epigenetic mechanisms, such as chromatin remodeling, or by influencing DNA repair and genomic stability, or through transcriptional reprogramming that regulates metabolic and signaling pathways? Future research must further dissect the multi-dimensional regulatory mechanisms of histone modifiers to provide substantial theoretical support for the precise intervention of age-related diseases (Sen et al., 2016; Yao et al., 2016; Kawarazaki and Fujita, 2021).

### 2.3 Regulatory network of non-coding RNAs

In the complex network of life regulation, non-coding RNAs (ncRNAs), a class of RNA molecules that do not encode proteins, have increasingly become a focal point of research in the field of aging epigenetics. The rapid advancement of high-throughput sequencing technologies and bioinformatics has continuously unveiled the diverse types and functions of ncRNAs. Their critical role in age-related diseases has become increasingly prominent, offering new perspectives for a deeper understanding of the molecular mechanisms of aging and the development of novel anti-aging strategies.

lncRNAs and miRNAs are two well-studied members of the ncRNA family. lncRNAs can regulate gene expression through various mechanisms. lncRNAs can regulate gene expression through various mechanisms. For instance, in neurons, the lncRNA UBE3A-ATS can silence the paternal UBE3A gene in cis, a mechanism that is significant in neurodevelopmental disorders such as Angelman syndrome (Wolter et al., 2020). Simultaneously, lncRNAs can dynamically regulate mRNA translation and degradation processes by forming ribonucleoprotein (RNP) condensates, a process that changes significantly during aging. Studies indicate that with age, the composition and function of RNP condensates alter, leading to the inhibited translation of specific mRNAs, thereby affecting cellular homeostasis and function (Pushpalatha et al., 2022). miRNAs primarily mediate the degradation or inhibition of translation of target mRNAs by binding to their 3′ untranslated regions (UTRs), achieving finely-tuned regulation of gene expression. During aging, miRNA expression profiles undergo significant changes, which are closely linked to aging-related phenotypes such as inflammation, metabolic disorders, and apoptosis (Sharma and Lu, 2018). For example, miR-346 plays a dual role in transcriptional activation and DNA damage repair, and its aberrant expression is closely associated with the development of age-related diseases such as prostate cancer (Fletcher et al., 2022). Furthermore, miRNAs can serve as potential biomarkers for monitoring the aging process and evaluating the effects of anti-aging interventions (Sharma and Lu, 2018; Butz et al., 2024).

In addition to these types, other non-coding RNAs (ncRNAs) such as circular RNAs (circRNAs) and small nucleolar RNAs (snoRNAs) play significant roles in the regulation of aging. CircRNAs can function as miRNA sponges or interact with RNA-binding proteins to modulate gene expression and signaling pathways (Butz et al., 2024). SnoRNAs are primarily involved in the modification and processing of ribosomal RNA (rRNA), and their functional disruption can compromise ribosome homeostasis, leading to cellular senescence (Wolter et al., 2020). For instance, the Snord115 gene cluster can influence the silencing state of the UBE3A gene in neurons by regulating the expression of UBE3A-ATS, offering a novel therapeutic target for Angelman syndrome. NcRNAs can affect gene expression patterns and cell fate decisions by regulating DNA methylation, histone modifications, and chromatin conformation. For example, the long non-coding RNA (lncRNA) ncRNA-a3 can enhance the binding of the p300/BRG1 complex to the TAL1 gene locus, activating an erythroid-specific transcriptional program essential for red blood cell differentiation (Matur et al., 2025). Furthermore, ncRNAs can influence genomic stability and cellular senescence by forming RNA-DNA hybrids (R-loops) and regulating transposon activity (Ostrowski et al., 2018). Studies suggest that the Pbp1/ATXN2 protein can inhibit the accumulation of RNA-DNA hybrids, thereby maintaining the stability of ribosomal DNA (rDNA) repeat sequences and delaying cellular senescence.

NcRNAs show great potential in the treatment of age-related diseases. RNA therapies based on miRNAs and lncRNAs hold broad prospects in areas such as cancer, CVDs, and neurodegenerative diseases (Winkle et al., 2021; Shah and Giacca, 2022). By designing specific antisense oligonucleotides (ASOs) or RNA mimics, researchers can precisely modulate the expression and function of ncRNAs to intervene in disease progression (Winkle et al., 2021). Additionally, ncRNAs can serve as liquid biopsy biomarkers for the non-invasive monitoring of individual health status and aging progression (Li et al., 2023; Butz et al., 2024). However, current research on ncRNAs in the context of aging epigenetics faces numerous challenges. The diverse functions and complex mechanisms of ncRNAs are difficult to fully decipher; their expression and function are highly tissue- and context-specific, which complicates precise clinical regulation (Anastasiadou et al., 2018; Sharma and Lu, 2018). Furthermore, the interaction network between ncRNAs and other epigenetic modifications remains incompletely understood, limiting comprehensive insights into their overall role in aging (Anastasiadou et al., 2018).

In summary, ncRNAs play multiple roles in the epigenetic regulation of aging. Related research not only uncovers the molecular mysteries of aging but also offers innovative ideas for developing anti-aging intervention strategies. In the future, technological advancements and deeper investigations will further explore the potential of ncRNAs in diagnosing and treating age-related diseases, providing robust support for the goal of promoting healthy human longevity. These research findings clearly demonstrate that miRNAs not only play a crucial role in the aging process and its associated pathologies but also represent significant potential targets for delaying aging and treating age-related diseases.

### 2.4 Degradation of chromatin three-dimensional structure (chromatin remodeling): heterochromatin loss, laminopathy, and genomic instability

The degradation of the three-dimensional (3D) chromatin structure, commonly referred to as chromatin remodeling, represents a fundamental research direction within the field of aging epigenetics. By modifying the spatial organization patterns of chromatin, this process directly regulates gene expression programs, thereby influencing cellular senescence and the functional decline of organs. Core features of chromatin remodeling include the loss of heterochromatin and lamin dysfunction. Collectively, these alterations drive genomic instability, serving as key epigenetic contributors to aging and related diseases (Yusufova et al., 2021; Zhang et al., 2021).

Heterochromatin, characterized as a highly condensed and transcriptionally silent region of chromatin, experiences loss as a hallmark event in aging-related chromatin remodeling. It is categorized into facultative heterochromatin, marked by H3K27me3, and constitutive heterochromatin, marked by histone H3 lysine 9 trimethylation (H3K9me3). Both types undergo differential remodeling during aging: facultative heterochromatin tends to transition from the repressive B compartment to the transcriptionally active A compartment, while constitutive heterochromatin exhibits enhanced self-interactions (Zhang et al., 2021). This compartment switching is accompanied by a significant increase in chromatin accessibility, leading to “leaky expression” of originally silent genes, such as early developmental genes and repetitive elements. Concurrently, increased chromatin accessibility at CTCF binding sites in aging cells induces the formation of new loop structures, further exacerbating three-dimensional genome disorganization (Zhang et al., 2021). The regulation of heterochromatin loss is influenced by various chromosomal proteins and remodeling factors, including heterochromatin protein 1a (HP1a), Polycomb group proteins, and the prolyl isomerase PIN1. The loss of HP1a function has been shown to shorten lifespan in invertebrates, while its overexpression can extend both healthspan and lifespan. PIN1, recognized as a conserved factor for heterochromatin stability across species, exhibits that its deficiency leads to progeroid symptoms and neurodegenerative pathologies in organisms ranging from flies to mammals. Conversely, maintaining PIN1 function has been demonstrated to delay heterochromatin relaxation (Zhang et al., 2021). Furthermore, abnormal histone modifications that occur during aging, such as increased dimethylation of histone H3 at lysine 36 (H3K36me2) and decreased trimethylation of H3 at lysine 27 (H3K27me3), facilitate the transition of chromatin from a compact to a relaxed state. This transition accelerates heterochromatin disintegration, subsequently unlocking the expression of stem cell-related genes and heightening the risk of malignant transformation in senescent cells (Yusufova et al., 2021).

Lamin abnormalities represent a critical aspect of the degradation of 3D chromatin structure. The nuclear lamina, a supportive structure located at the inner nuclear membrane, serves to anchor heterochromatin and specific genomic regions. Dysfunction of this structure disrupts the spatial association between chromatin and the nuclear envelope, leading to the detachment and mislocalization of heterochromatin from the lamina. Although specific literature on lamins is not directly cited here, it is evident that lamin abnormalities and heterochromatin loss have synergistic effects based on the principles of 3D chromatin structure regulation. Together, these factors contribute to blurred compartment boundaries and disintegration of TAD structures, consequently causing dysregulation of gene transcription programs (e.g., aberrant activation of SASP genes), and accelerating cellular senescence (Yusufova et al., 2021; Zhang et al., 2021).

The direct consequence of 3D chromatin structure degradation is the exacerbation of genomic instability. On one hand, the loss of heterochromatin activates repetitive elements (e.g., LTR retrotransposons, satellite sequences), whose aberrant amplification can disrupt genomic integrity (Zhang et al., 2021). On the other hand, disruptions in chromatin structure (e.g., TAD disintegration, compartment fusion) hinder the recruitment of DNA damage repair factors (e.g., BRCA1, 53BP1) to damage sites, leading to the accumulation of DNA damage and further worsening genomic stability (Yusufova et al., 2021). For example, chromatin relaxation in aging cells increases the exposure of DNA to damaging agents, while dysfunctional repair mechanisms prevent the timely clearance of damage, forming a vicious cycle of “structural disorder - damage accumulation - accelerated aging” (Yusufova et al., 2021; Zhang et al., 2021).

The degradation of 3D chromatin structure is closely associated with the development of age-related diseases. In the brains of AD patients, blurred chromatin compartment boundaries and the fusion of adjacent chromatin domains are considered hallmark events of accelerated brain aging. This structural abnormality directly leads to the downregulation of key functional genes, such as synaptic maintenance genes, in neurons, thereby exacerbating neurodegenerative pathology (Oh et al., 2016); In lymphoma, mutations in histone H1 cause large-scale chromatin decompaction, activating early developmental genes through epigenetic reprogramming, which ultimately drives tumorigenesis (Yusufova et al., 2021). These findings indicate that the degradation of 3D chromatin structure is both a molecular feature of aging and a core epigenetic mechanism in disease pathogenesis.

Technological breakthroughs have facilitated in-depth analyses of the mechanisms underlying 3D chromatin structure degradation. Single-cell chromatin conformation capture techniques, such as Droplet Hi-C, enable the mapping of 3D genome architectures in individual cells within heterogeneous tissues, thereby precisely capturing dynamic changes in chromatin structure during aging (Chang et al., 2024); Additionally, deep learning models like C.Origami can predict 3D chromatin structures from DNA sequence and chromatin accessibility data, offering new tools for high-throughput screening of key factors regulating chromatin remodeling (Tan et al., 2023). Despite significant advancements, several key challenges persist. First, the causal relationships between chromatin remodeling factors, such as HP1a and PIN1, and lifespan regulation in mammals remain inadequately established, particularly as experimental evidence for lifespan extension in vertebrates through the enhancement of these factors is still lacking. Second, the molecular interaction mechanisms linking laminopathy and chromatin structure degradation require further investigation. Third, reversing the aging process and ameliorating age-related diseases by targeting the 3D chromatin structure necessitates more translational medical research.

In conclusion, the degradation of the three-dimensional chromatin structure, characterized by heterochromatin loss, laminopathy, and genomic instability, is a central aspect of epigenetic regulation in aging. Research into its mechanisms not only deepens our understanding of the molecular essence of aging but also identifies potential targets for epigenetic intervention in age-related diseases, such as neurodegenerative disorders and cancer. Future efforts should prioritize the conserved functions of chromatin remodeling factors across species, the structure-function regulatory network, and the translation of interventional strategies, thereby providing theoretical support for the achievement of healthy aging.

### 2.5 Suppression of retrotransposons

Retrotransposons, particularly Long Interspersed Nuclear Element-1 (LINE-1 or L1), play a pivotal role in the epigenetics of aging. These elements are extensively distributed throughout the mammalian genome, constituting approximately 17% of the human genome (Ramini et al., 2022). They function through a “copy-and-paste” transposition mechanism, employing their self-encoded ORF1 and ORF2 proteins to facilitate reverse transcription and subsequent integration (Arancio, 2019). While retrotransposons have significantly contributed to genomic diversity and adaptability throughout evolution, their aberrant activation during aging and neurodegenerative processes is closely associated with DNA damage, mutations, and inflammatory responses (Ostrowski et al., 2018; Tan et al., 2018).

Dysregulation of epigenetic control serves as a significant trigger for the activation of retrotransposons during the aging process. As individuals age, global genomic DNA methylation levels typically decline, particularly in repetitive sequence regions such as LINE-1 and Alu elements (Wahl et al., 2021; Mustafin and Khusnutdinova, 2024; Mustafin and Khusnutdinova, 2024). In aged human umbilical vein endothelial cells (HUVECs) and skin fibroblasts (NHDFs), the RNA expression of LINE-1 and Alu elements is markedly upregulated, accompanied by a substantial increase in the copy number of retrotransposon DNA within the cytoplasm (Simon et al., 2019). This indicates that reverse transcription of retrotransposons occurs in senescent cells, generating DNA sequences that exacerbate genomic instability (Narisu et al., 2019). Additionally, alterations in chromatin structure play a crucial role in regulating retrotransposons. The reorganization and de-condensation of heterochromatin are characteristic features of aging cells, potentially leading to the reactivation of previously silenced retrotransposons (Pabis et al., 2024). For example, the SIRT6 protein mono-ADP-ribosylates KAP1, promoting its complex formation with HP1, which packages LINE-1 DNA into transcriptionally silent heterochromatin (Arribas et al., 2024). SIRT6 knockout mice exhibit significant activation of LINE-1, accompanied by increased genomic instability and DNA damage, further confirming the association between retrotransposon activity and age-related pathology (Lanciano and Cristofari, 2020).

Recent research on suppressing retrotransposons has opened new avenues for delaying aging and treating related diseases. The use of antiretroviral drugs or RNA interference techniques to inhibit LINE-1 activity has been shown to significantly alleviate the progeroid phenotype in SIRT6 knockout mice (Author Anonymous, 2023). Epigenetic drugs, such as DNMT inhibitors and HDAC inhibitors, can re-silence retrotransposons, thereby reducing their mediated genomic damage and inflammatory responses (Author Anonymous, 2024). However, research on retrotransposon suppression continues to face numerous challenges. The mechanisms underlying their activation are complex, involving the coordinated action of various epigenetic and chromatin regulatory factors (Oomen et al., 2025). Furthermore, the activity of retrotransposons exhibits tissue-specific variations; for instance, in neurons, their activity differs significantly compared to other tissues, complicating research efforts (Handa et al., 2025). In Huntington’s disease (HD) mouse models, an increased LINE-1 copy number and transcriptional levels in brain tissue are closely associated with disease progression, while similar phenomena are not observed in other tissues (Jang et al., 2024). His suggests a need for in-depth investigation into the functional differences and tissue-specific regulatory mechanisms.

Recent studies have further elucidated the role of retrotransposition in the aging of complex metazoans, including humans. Retrotransposons, consisting of LINEs and Short Interspersed Nuclear Elements (SINEs), are reactivated in senescent cells and throughout the lifespan, exerting detrimental effects through genetic and epigenetic changes or by activating immune pathways (Ostrowski et al., 2018). Mechanistically, the epigenetic derepression of LINE-1 RNA inhibits the epigenetic reader Suv39H1, resulting in a global reduction in H3K9me3 and heterochromatin. Concurrently, double-stranded cDNA produced by the reverse transcription of LINE-1 RNA activates the cyclic GMP-AMP synthase/stimulator of interferon genes (cGAS/STING)/interferon pathway. Treatment with nucleoside reverse transcriptase inhibitors (NRTIs) can suppress or attenuate retrotransposition, thereby extending the lifespan and improving the healthspan of Sirt6-deficient mice, while ameliorating skeletal and muscle phenotypes. Furthermore, treating aged wild-type mice with NRTIs reduces levels of DNA damage markers (Ostrowski et al., 2018). *In vivo* antisense oligonucleotide (ASO) therapy targeting retrotransposons can also extend the lifespan of progeroid mice. Notably, rare SIRT6 variants found in centenarians exhibit stronger suppression of LINE-1 retrotransposons, enhanced genomic stability, and are more effective than wild-type SIRT6 in inducing apoptosis in cancer cells. These findings indicate a causal role for retrotransposons in aging, suggesting that interventions targeting their activity may hold promise for extending healthspan. Subsequent clinical research focusing on different functional retrotransposons may provide new intervention strategies for aging and related pathologies.

### 2.6 Gene expression changes

The core mechanisms of epigenetic regulation ultimately converge on the dynamic modulation of gene expression levels. During aging, the intracellular transcriptional regulatory network gradually becomes imbalanced, and transcriptional noise significantly increases, leading to aberrant synthesis and maturation defects of numerous mRNAs. Through the three core mechanisms of DNA methylation, histone modifications, and non-coding RNA (ncRNA) regulation, the cellular transcriptional network is reshaped, triggering a decline in cellular function and an imbalance in tissue homeostasis. Simultaneously influenced by environmental factors and technological innovations, these changes provide key insights for elucidating the molecular mechanisms of aging and for intervening in age-related diseases (Anand Brown et al., 2015; Brinkmeyer-Langford et al., 2016; Balliu et al., 2019).

Environmental factors and technological advancements further enrich the research dimensions of gene expression regulation in aging. Environmental factors can reshape gene regulatory networks by inducing changes in DNA methylation patterns and adjustments in histone modification states, thereby accelerating the progression of the epigenetic clock. Meanwhile, the application of microarray technology, single-cell transcriptomics, plasma proteomics, and epigenome editing techniques—such as CRISPR-dCas9-mediated CRISPR activation/inhibition (CRISPRa/CRISPRi)—provides high-resolution tools for analyzing gene expression changes (Tettey et al., 2023; Hunt et al., 2025). For example, single-cell sequencing reveals cellular heterogeneity and individual differences in gene expression during aging. CRISPR-dCas9 can simulate age-related methylation changes and validate their impact on gene expression. Furthermore, single-cell transcriptomic studies of multiple organs in mice have found that aging is accompanied by gene expression remodeling, which significantly affects inflammatory responses, protein folding, extracellular matrix homeostasis, and mitochondrial function. Concurrently, the decreased efficiency of transcriptional and post-transcriptional regulation leads to proteostasis imbalance, offering new directions for developing anti-aging strategies.

The dysregulation of epigenetic control over gene expression is intricately linked to age-related diseases. In AD, genes associated with synaptic plasticity and neuronal function are downregulated due to promoter hypermethylation, which exacerbates neuronal decline. In cancer, tumor suppressor genes are inactivated through epigenetic silencing, thereby promoting tumorigenesis and disease progression (Esteller, 2007; Li et al., 2019; Gao et al., 2022).

In summary, the epigenetic regulatory mechanisms governing gene expression during aging involve the synergistic action of multi-dimensional modifications and are influenced by both environmental factors and technological advancements. Future research should focus on further analyzing the complexity of epigenetic regulatory networks and exploring precision intervention strategies based on epigenetic editing. This will provide theoretical support and a translational basis for delaying aging and preventing or treating age-related diseases (Wei et al., 2021; Shireby et al., 2022).

## 3 Epigenetic progress and treatment of age-related diseases

**FIGURE 2:**
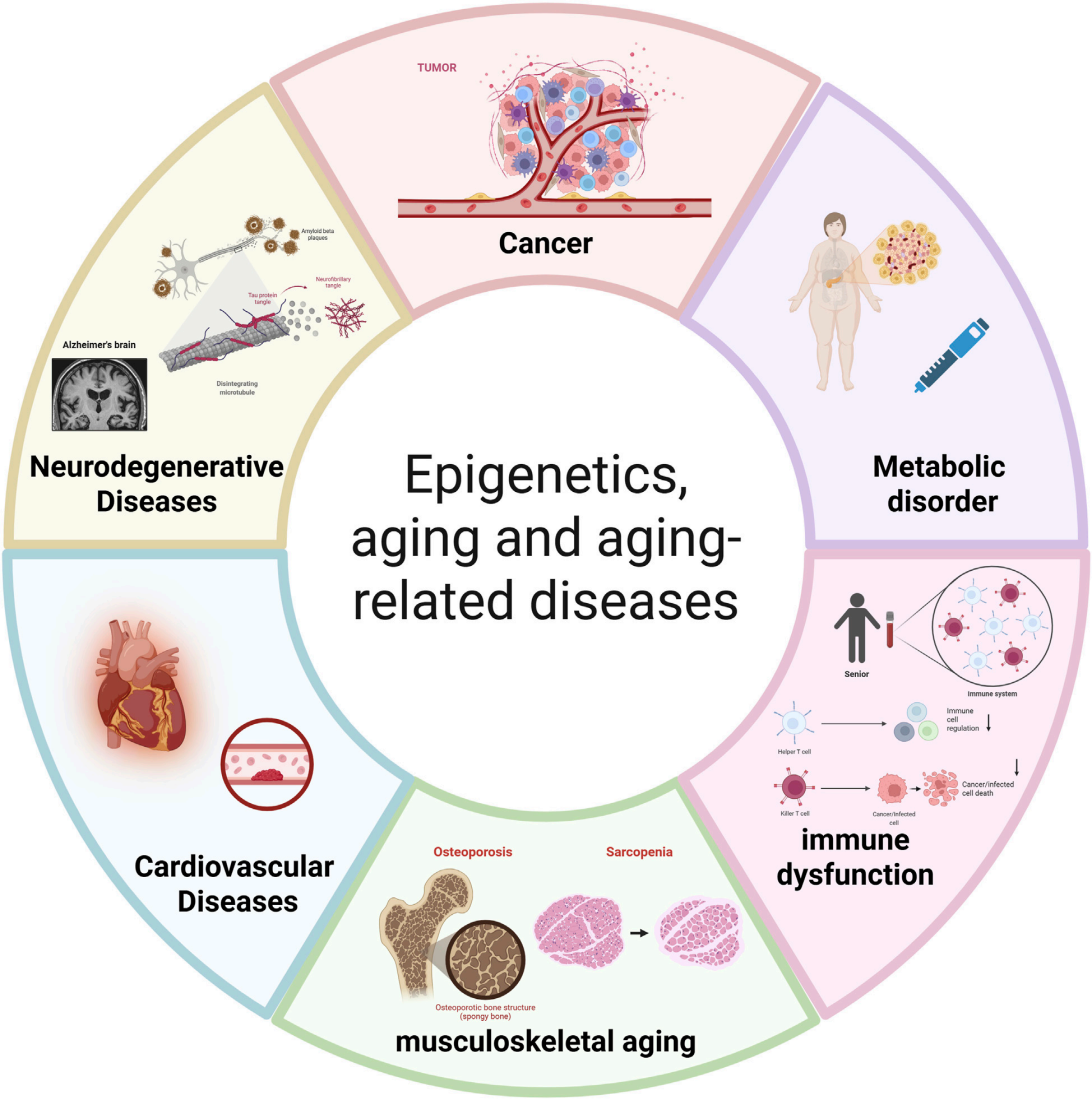
The central role of epigenetic dysregulation in aging and age-related diseases. This diagram illustrates how age-associated epigenetic alterations drive the pathogenesis of major disease categories: Cancer: Driven by global hypomethylation and promoter hypermethylation of tumor suppressor genes (e.g., p16INK4a, RASSF1A). Metabolic disorders (e.g., T2DM): Associated with methylation changes in insulin signaling genes (e.g., INS promoter) and HDAC overexpression. Immune dysfunction: Involving DNA methylation changes in immune genes (e.g., IL-7Rα) and histone modification imbalances. Musculoskeletal aging (e.g., Osteoporosis, Sarcopenia): Linked to methylation of osteogenic genes (e.g., RUNX2, SOST) and miRNA dysregulation (e.g., miR-29). Cardiovascular diseases: Influenced by methylation of vascular genes (e.g., NOS3, ABCA1) and HDAC-mediated chromatin remodeling. Neurodegenerative diseases (e.g., Alzheimer’s, Parkinson’s, Huntington’s disease): Associated with DNA methylation abnormalities in synaptic genes, histone deacetylation, and non-coding RNA dysregulation (la Torre et al., 2023). Abbreviations: LINE-1, Long Interspersed Nuclear Element-1; p16INK4a, Cyclin-Dependent Kinase Inhibitor 2A; RASSF1A, Ras Association Domain Family Member 1A; INS, Insulin; HDAC, Histone Deacetylase; PPARγ, Peroxisome Proliferator-Activated Receptor γ; IL-7Rα, Interleukin-7 Receptor α; IFN-γ, Interferon γ; RUNX2, Runt-Related Transcription Factor 2; SOST, Sclerostin; miR-29, microRNA-29; NOS3, Nitric Oxide Synthase 3; ABCA1, ATP-Binding Cassette Sub-Family A Member 1; SERCA2a, Sarcoplasmic Reticulum Calcium ATPase 2a; BDNF, Brain-Derived Neurotrophic Factor; miR-132, microRNA-132. (Created with BioRender.com).

In the initiation and progression of aging and age-related diseases, epigenetics plays an irreplaceable and critical role. It finely regulates gene expression and cellular function primarily through DNA methylation, various histone modifications (including acetylation, ubiquitination, and methylation), and non-coding RNA regulatory mechanisms. As research deepens, accumulating evidence indicates that diseases such as cancer, metabolic disorders like T2DM, (CVDs), neurodegenerative diseases, and immune dysfunction are significant consequences of organismal aging, resulting from the interplay of genetic and environmental factors (Figure 2). The occurrence of these diseases fundamentally stems from complex molecular changes; dysregulation of epigenetic modifications disrupts the body’s original homeostatic balance, thereby triggering disorders and declines in cellular physiological functions. Furthermore, epigenetic changes are theoretically reversible, thus offering opportunities for the design of novel anti-aging therapies. The following sections explore the specific regulatory mechanisms and pathways of epigenetics in aging-related diseases from the perspective of different disease types, revealing its key impact on disease pathology.

### 3.1 Neurodegenerative diseases

Aging is a core pathogenic factor in various neurodegenerative diseases, including AD, PD, and Huntington’s Disease (HD). As research progresses, the crucial role of epigenetic regulatory mechanisms in the development of these diseases has become increasingly evident, giving rise to the emerging field of “neuroepigenetics.” DNA methylation, a key component of epigenetic regulation, is not only involved in gene silencing but also plays a significant role in memory formation. Among the DNMTs, DNMT1, DNMT3A, and DNMT3D are particularly influential in maintaining normal brain physiological function.

The core pathology of PD is characterized by the loss of dopaminergic neurons in the substantia nigra and the aggregation of alpha-synuclein (SNCA). The epigenetic regulation of PD primarily focuses on the methylation imbalance of the SNCA gene and the inhibition of histone acetylation. Studies have identified hypermethylation in the promoter region of the SNCA gene in patients with alcohol-dependent anorexia, which results in downregulated expression. However, the inhibition of DNA methyltransferase (DNMT) activity significantly restores SNCA gene expression, suggesting that DNA methylation fine-tunes SNCA protein levels (Antico et al., 2025). Furthermore, abnormally aggregated alpha-synuclein can directly bind to histones, inhibiting histone acetyltransferase activity and further compacting chromatin structure through protein-histone interactions. This process suppresses the expression of neuroprotective genes while exacerbating mitochondrial dysfunction. Epigenetic interventions, such as HDAC inhibitors, can restore histone acetylation levels, activate mitophagy-related genes (e.g., PINK1, Parkin), and improve mitochondrial homeostasis in PD models (Nixon and Rubinsztein, 2024; Antico et al., 2025).

AD is characterized by progressive neuronal loss in the cerebral cortex and hippocampus, aberrant β-amyloid (Aβ) deposition, hyperphosphorylation of tau protein, and cognitive decline. The pathological process of AD is closely associated with aging-driven epigenetic disruptions. Specifically, phosphorylation and hyperphosphorylation of histone H3, along with deacetylation of histone H4, have been observed in the hippocampal tissue of early-stage AD patients; these epigenetic modifications directly inhibit the transcription of genes related to synaptic plasticity, such as brain-derived neurotrophic factor (BDNF) (Shvetcov et al., 2025). Concurrently, significant DNA methylation abnormalities have been identified in genes associated with synaptic function and neuroinflammation, including the regulatory pathway of apolipoprotein E ε4 (APOEε4), which further exacerbates neuroinflammatory responses and neuronal damage (Shvetcov et al., 2025). In terms of therapeutic interventions, HDAC inhibitors demonstrate notable neuroprotective potential. For instance, the HDAC6 inhibitor PB118 can facilitate the clearance of Aβ deposits by upregulating phagocytosis, enhance the microtubule network by increasing acetylation of α-tubulin levels, significantly reduce levels of phosphorylated tau (p-tau) associated with AD, regulate the expression of inflammatory factors and chemokines, and repair dysfunctions in the autophagy-lysosomal pathway (Wang and Cui, 2016; Nixon and Rubinsztein, 2024).

Huntington’s Disease (HD) is caused by the abnormal expansion of CAG repeats in the huntingtin gene (HTT). The presence of mutant huntingtin protein (mHTT) leads to dysregulated transcription, mitochondrial dysfunction, and neuronal apoptosis, with an imbalance in epigenetic modifications serving as a key driver of its pathological progression. Research utilizing model organisms and human samples has demonstrated global alterations in DNA methylation levels in the brains of HD patients, alongside significant imbalances in the acetylation modifications of histones H3K9 and H3K14. mHTT has been shown to bind to histone deacetylases (HDACs), enhancing their activity, which results in the transcriptional repression of autophagy-related genes such as ATG5 and LC3, thereby exacerbating mHTT protein aggregation (Nixon and Rubinsztein, 2024). Concurrently, the aberrant regulation of Polycomb Repressive Complex 2 (PRC2) by mHTT leads to the abnormal accumulation of the repressive mark H3K27me3 on neural differentiation genes, further accelerating degenerative changes in dopaminergic neurons (Cederquist et al., 2020).

In summary, aging-related epigenetic dysregulation represents a fundamental pathological mechanism in neurodegenerative diseases. This perspective enhances our understanding of disease mechanisms and informs treatment development. Future research should aim to further integrate multi-omics data, including epigenomic, transcriptomic, and proteomic information. Additionally, a deeper analysis of epigenetic regulatory networks is essential. Promoting the translation of epigenetically targeted drugs from basic research to clinical applications will offer novel strategies for the prevention and treatment of aging-related neurodegenerative diseases.

### 3.2 Cardiovascular diseases

CVDs are the leading global cause of death and disease burden, with a significant association with the aging process. The imbalance of epigenetic homeostasis—characterized by disordered DNA methylation patterns, abnormal histone modifications, and ncRNA control—serves as a critical molecular link between aging and cardiovascular pathology. This imbalance dynamically regulates gene expression programs in heart and vascular cells, including cardiomyocytes, vascular smooth muscle cells, and leukocytes. It disrupts cardiovascular homeostasis and drives the development of diseases such as atherosclerosis, heart failure, and coronary artery disease (CAD), while also presenting epigenetic targets for diagnosis and targeted therapy (Das et al., 2020; Monisha et al., 2023; Bingyu et al., 2024).

During aging, the synergistic functional imbalance between DNMTs and the ten-eleven translocation 2 (TET2) enzyme serves as an initiating event in cardiovascular epigenetic disruption. This imbalance between DNMT-catalyzed methylation and TET2-mediated 5-methylcytosine hydroxylation disrupts genomic methylation homeostasis, particularly impacting the expression of key genes essential for cardiovascular function. For instance, mutations in the leukemia precursor gene DNMT3A, extra sex combs-like genes, and TET2 alter the inflammatory properties of leukocytes, promote the release of pro-inflammatory factors, and accelerate atherosclerotic plaque formation (Das et al., 2020). In terms of lipid metabolism, age-related aberrant methylation of the carnitine palmitoyltransferase 1A (CPT1A) gene directly leads to an imbalance in blood lipoprotein and triglyceride metabolism, while hypermethylation of the ATP-binding cassette subfamily A member 1 (ABCA1) gene impedes reverse cholesterol transport, significantly contributing to hypercholesterolemia and atherosclerosis (Shi et al., 2017; Das et al., 2020). Furthermore, methylation changes in genes such as nitric oxide synthase 3 (NOS3, which regulates vasodilation), apolipoprotein E (APOE, involved in lipid transport), v-rel avian reticuloendotheliosis viral oncogene homolog A (RELA, which regulates inflammation), and Kruppel-like factor 4 (KLF4, which maintains vascular homeostasis) all play crucial roles in the initiation and progression of atherosclerosis (Das et al., 2020; Bingyu et al., 2024). In atherosclerotic lesions, the abnormal proliferation and migration of vascular smooth muscle cells are also regulated by the methylation of genes such as type XV collagen alpha 1 and transforming growth factor beta receptor III, while dysfunction of the chromatin remodeling complex BAF (SWI/SNF) further exacerbates pathological vascular wall remodeling (Das et al., 2020; Bingyu et al., 2024).

The age-related imbalance in histone modifications, particularly acetylation, is closely associated with the aberrant expression and function of histone deacetylases (HDACs), which directly contributes to cardiovascular inflammation, a decline in myocardial function, and vascular pathology. Research indicates that the expression profiles of HDAC family members undergo significant changes with aging; for instance, polymorphisms in the HDAC9 gene can elevate the risk of large vessel atherosclerotic stroke (LVAS) by modulating the expression of genes involved in inflammation, lipid metabolism, and platelet aggregation (Shroff et al., 2019). Furthermore, the upregulation of HDAC1, HDAC2, and HDAC4 in peripheral blood mononuclear cells from patients with coronary artery disease (CAD) shows a significant correlation with critical functional parameters such as cardiac ejection fraction and diastolic function, suggesting their potential utility as biomarkers for CAD diagnosis and severity assessment (Monisha et al., 2023). Mechanistically, HDACs alter chromatin structure by removing acetyl groups from histones, which inhibits the transcription of vasoprotective genes (e.g., endothelial nitric oxide synthase, anti-inflammatory interleukin-10 (IL-10)), while simultaneously activating pro-inflammatory pathways (e.g., nuclear factor-kappa B (NF-κB)) and genes associated with vascular smooth muscle cell proliferation, thereby accelerating the progression of atherosclerosis and myocardial remodeling (Davalos and Esteller, 2023; Monisha et al., 2023).

Age-induced alterations in the non-coding RNA (ncRNA) expression profile significantly affect cardiovascular cell function through post-transcriptional regulation and provide novel biomarkers for diagnosis. Specifically, decreased expression of miRNA-425 and miRNA-744 in cardiac fibroblasts is associated with an increased risk of cardiac fibrosis and heart failure. During myocardial ischemia, upregulation of miRNA-15 expression induces cardiomyocyte apoptosis by targeting anti-apoptotic genes, thereby exacerbating ischemic injury. In contrast, miRNA-204 and miRNA-34b exert vasoprotective effects by inhibiting genes related to vascular smooth muscle cell calcification, such as RUNX2 (Das et al., 2020; Bingyu et al., 2024). Regarding long non-coding RNAs (lncRNAs), the expression of specific lncRNAs, such as ANRIL, is significantly elevated in the myocardial tissue of heart failure patients, with levels positively correlating with the degree of cardiac remodeling and the extent of reduced ejection fraction. This correlation makes ANRIL a potential marker for assessing disease progression and prognosis (Das et al., 2020; Furquim et al., 2023). Furthermore, lncRNAs can influence the expression of coronary artery disease (CAD) risk genes, including HCFC1 and RNF8, by regulating the recruitment of chromatin remodeling complexes such as BAF, thus linking aging with susceptibility to CVDs (Bingyu et al., 2024).

The reversibility of epigenetic modifications renders them ideal targets for interventions in age-related CVDs. Therapeutically, HDAC inhibitors exhibit significant vascular protective and myocardial repair potential. Specifically, the inhibition of HDAC activity restores histone acetylation levels, which can activate anti-inflammatory genes and promote the expression of vascular homeostasis genes. Additionally, this inhibition suppresses vascular smooth muscle cell proliferation and slows the progression of atherosclerosis (Shroff et al., 2019; Monisha et al., 2023). Additionally, DNMT inhibitors can reverse the aberrant hypermethylation of genes such as ABCA1 and NOS3, thereby improving cholesterol metabolism and vasodilation function (Das et al., 2020; Bingyu et al., 2024). In terms of diagnosis and prognosis assessment, the expression profiles of HDACs (e.g., HDAC1, HDAC2, HDAC4, and HDAC9), DNA methylation markers (e.g., methylation levels of CPT1A, ABCA1), and ncRNA expression levels (e.g., miRNA-15, lncRNA ANRIL) have proven useful for the early diagnosis and risk stratification of CAD and heart failure, thereby providing a theoretical basis for “epigenetic diagnosis” (Furquim et al., 2023; Monisha et al., 2023; Bingyu et al., 2024).

In conclusion, epigenetic regulation serves as a crucial mechanism for maintaining cardiac homeostasis throughout the aging process. Disruption of this regulatory network is closely associated with the development of various age-related CVDs. A comprehensive investigation of epigenetic mechanisms will yield new strategies and targets for the prevention and treatment of CVDs.

### 3.3 Metabolic syndrome

Metabolic Syndrome (MetS), a prevalent and complex condition associated with aging, serves as a core risk factor for chronic diseases such as CVD and T2DM. Its incidence significantly increases with age, posing a serious threat to the health of the elderly population (Anand Brown et al., 2015). Recent studies have confirmed that the imbalance of epigenetic homeostasis constitutes a key molecular link between aging and the pathological processes of MetS. Aging-induced disruptions in DNA methylation patterns, abnormal histone modifications, and dysregulated ncRNA control dynamically regulate the expression of metabolism-related genes. These disruptions compromise insulin signaling, lipid metabolism, and inflammatory homeostasis, ultimately driving the development of MetS. This understanding offers new perspectives for elucidating disease mechanisms and developing targeted interventions.

During aging, the synergistic functional imbalance between DNMTs and demethylases, such as the TET family, results in abnormal genomic methylation patterns that directly affect metabolic pathways related to MetS. In patients with MetS, key genes involved in the insulin signaling pathway, including insulin receptor substrate genes, are downregulated due to aberrant methylation in peripheral blood cells and adipose tissue—the primary tissue for age-related adipocyte metabolic abnormalities—leading to insulin resistance. Furthermore, methylation changes in genes associated with lipid metabolism, such as those involved in lipoprotein metabolism, directly disrupt lipid homeostasis, exacerbating typical MetS phenotypes like hypertriglyceridemia and low levels of high-density lipoprotein cholesterol (Beddows et al., 2024). Additionally, 5-hydroxymethylcytosine (5hmC), a crucial intermediate in the DNA demethylation process, plays a more significant regulatory role in age-related cardiometabolic diseases compared to 5-methylcytosine (5 mC). For example, in the context of MetS-related cardiovascular injury, 5hmC enhances energy supply by regulating the expression of myocardial metabolic genes, indicating its potential therapeutic value (Tabish et al., 2019).

The role of DNA methylation abnormalities is more clearly defined in T2DM, a common complication of MetS. Age-related methylation of nine CpG islands located upstream of the human insulin gene promoter can directly inhibit the expression of reporter genes driven by this promoter. This disruption affects the balance of insulin and glucagon secretion, accelerating the onset of hyperglycemia and the progression of T2DM. These findings underscore the central role of DNA methylation imbalance in metabolic disorders.

The age-related imbalance in histone modifications, particularly acetylation and methylation, characterized by the overactivation of histone deacetylases (HDACs), regulates metabolic inflammation and insulin sensitivity by reshaping chromatin structure. Studies indicate that HDAC activity significantly increases with age in the adipose tissue and liver of patients with MetS. On one hand, this process compresses chromatin by removing acetyl groups from histones, thereby suppressing the transcription of anti-inflammatory genes (e.g., IL-10) and metabolic protective genes (e.g., adiponectin). On the other hand, it exacerbates adipose tissue inflammation and hepatic lipid deposition by modulating transcription factor activity (e.g., NF-κB), creating a vicious cycle of “inflammation-insulin resistance-metabolic disorder” (Beddows et al., 2024). Conversely, the inhibition of HDAC activity can restore histone acetylation levels, activate metabolic protective pathways, and enhance insulin sensitivity and lipid metabolism, thereby providing a clear target for intervention in MetS (Beddows et al., 2024).

Age-induced reshaping of the ncRNA expression profile affects metabolic pathways related to MetS through post-transcriptional regulation, providing potential diagnostic markers. In the context of miRNAs, the aberrant expression of miR-30c-1 is closely associated with cardiovascular complications linked to MetS, such as myocardial hypertrophy and heart failure. This miRNA disrupts energy supply by targeting myocardial metabolic genes, including glucose transporter 4 (GLUT4), which exacerbates cardiac functional impairment (Tabish et al., 2019). Furthermore, age-related downregulation of miRNAs that regulate the insulin signaling pathway, such as miR-143/145, leads to increased degradation of insulin receptor substrate proteins, further aggravating insulin resistance. Regarding lncRNAs and circRNAs, these molecules participate in processes such as adipocyte differentiation and hepatic gluconeogenesis by acting as miRNA sponges or interacting with RNA-binding proteins; however, their specific regulatory mechanisms in the context of aging still require comprehensive analysis (Tabish et al., 2019).

The reversibility of epigenetic modifications presents ideal targets for the treatment of MetS. Preclinical studies indicate that DNA methyltransferase inhibitors (DNMTis), such as RG108, can partially restore cardiac energy metabolism and contractile function in models of pressure overload-induced cardiac hypertrophy associated with MetS by reversing the abnormal methylation of myocardial metabolic genes (Tabish et al., 2019). Histone deacetylase inhibitors (HDACis) improve adipose tissue inflammation and hepatic insulin resistance, while also reducing blood glucose and lipid levels in MetS patients by inhibiting HDAC activity (Beddows et al., 2024). Furthermore, the advent of CRISPR/Cas9 gene editing technology allows for precise modifications of epigenetic loci related to MetS, such as insulin promoter CpG islands and HDAC regulatory regions, thus providing technical support for “personalized epigenetic intervention” (Tabish et al., 2019). Drugs (e.g., DNMTis, HDACis) and technologies (e.g., CRISPR/Cas9) that are based on epigenetic mechanisms offer novel strategies for the precise treatment of MetS. Future research should concentrate on elucidating the mechanisms linking aging, epigenetics, and metabolism, addressing challenges such as tissue specificity and drug safety, and facilitating the translation of epigenetic interventions from preclinical studies to clinical applications, thereby providing new breakthroughs in the prevention and treatment of age-related MetS.

### 3.4 Cancer

Aging is the primary risk factor for cancer development, with a fundamental connection established through the core association of “cellular damage accumulation - genomic instability - epigenetic imbalance.” During the aging process, various environmental factors, including radiation, stress, and poor dietary habits, induce oxidative stress, resulting in DNA damage, such as single and double-strand breaks, as well as telomere shortening. The dynamic imbalance of epigenetic regulation not only serves as a critical driver of the age-related decline in genomic stability but also constitutes a fundamental molecular mechanism that facilitates the malignant transformation of normal cells (Davalos and Esteller, 2023; Laisné et al., 2025). Epigenetic modifications, including DNA methylation, histone modifications, and non-coding RNA regulation, reversibly modulate gene expression programs, contributing to the functional decline of senescent cells while also playing pivotal roles in tumor initiation, progression, and the development of drug resistance. This provides a cohesive perspective for understanding the pathology and intervention strategies that link aging and cancer (Davalos and Esteller, 2023; Sabzevari et al., 2025).

During aging, abnormal DNMT activity, such as decreased DNMT1 expression, leads to disordered methylation patterns characterized by “global hypomethylation and local hypermethylation,” which directly increases cancer susceptibility. On one hand, global hypomethylation—particularly in repetitive sequences like satellite DNA and long interspersed nuclear elements—weakens genomic stability, activates transposon activity, causes DNA insertion/deletion mutations, and accelerates tumor transformation. Sheaffer et al. confirmed in the ApcMin/+ mouse model that the complete loss of DNMT1 significantly accelerates intestinal cancer progression, corroborating the role of hypomethylation in tumorigenesis (Davalos and Esteller, 2023). On the other hand, hypermethylation of tumor suppressor gene promoter regions leads to gene silencing, which is an important driving event in aging-related cancers. For example, promoter hypermethylation of p16INK4a and TIMP3 is closely related to the development of oropharyngeal cancer; the methylation frequency of RASSF1A and GSTP1 promoters is significantly higher in hepatocellular carcinoma cells compared to normal cells; and hypermethylation of tumor suppressor genes in colorectal cancer not only drives the disease but also serves as a biomarker for early diagnosis (Davalos and Esteller, 2023). This methylation imbalance accumulates with age, gradually rendering senescent cells into an “epigenetically susceptible” state, paving the way for cancer development.

The age-related imbalance in histone modifications, specifically methylation and acetylation, disrupts gene expression regulatory networks by reshaping chromatin structure, which is directly linked to cancer development. In terms of modification patterns, levels of H3K4me3 decrease in senescent cells, disrupting histone methylation homeostasis and impairing both genomic integrity and transcriptional precision. Concurrently, mutations or aberrant activation of histone acetyltransferase (HAT), such as EZH2, contribute to the development of various cancers, including lymphoma and breast cancer, by catalyzing the abnormal accumulation of the repressive mark H3K27me3 on tumor suppressor genes (Davalos and Esteller, 2023; Sabzevari et al., 2025). Regarding acetylation, the balance between HAT and HDAC activity is disrupted with age. Increased HDAC activity results in histone hypoacetylation, which suppresses the expression of tumor suppressor genes, such as p53. Additionally, abnormal HAT activity may enhance the transcription of proto-oncogenes, such as MYC. Collectively, these changes promote the transformation of senescent cells into a malignant phenotype (Sabzevari et al., 2025).

DNA methylation-based epigenetic markers have emerged as crucial tools for diagnosing aging-related cancers. The detection of methylation signatures, such as the hypermethylation of specific tumor suppressor genes in colorectal cancer, within circulating tumor DNA (ctDNA) facilitates non-invasive early cancer screening and monitoring of treatment responses. This method demonstrates accuracy and specificity that significantly surpass traditional markers (Sabzevari et al., 2025). Furthermore, abnormal patterns of histone modifications (e.g., H3K4me3, H3K27me3) can also serve as prognostic indicators for cancer; for instance, the loss of H3K27me3 is associated with shorter survival in lung cancer patients (Baca et al., 2023).

The reversibility of epigenetic modifications positions them as promising targets for the treatment of aging-related cancers, with several drugs currently in clinical use. DNMT inhibitors, such as Azacitidine and Decitabine, counteract the hypermethylation of tumor suppressor genes, thereby restoring their expression. These agents have gained approval for the treatment of aging-related myelodysplastic syndromes (MDS) and acute myeloid leukemia (Davalos and Esteller, 2023). Similarly, HDAC inhibitors, including Vorinostat and Romidepsin, inhibit histone deacetylase activity, restore histone acetylation levels, and activate tumor suppressor genes, demonstrating significant efficacy in the treatment of peripheral T-cell lymphoma (PTCL) and marking a considerable advancement in the management of T-cell malignancies (Sabzevari et al., 2025). Furthermore, combination strategies that incorporate epigenetic drugs with immunotherapy—such as HDAC inhibitors enhancing the efficacy of PD-1 inhibitors—are currently in clinical trials, offering new avenues for the targeted treatment of aging-related cancers (Sabzevari et al., 2025).

### 3.5 Epigenetics and immunosenescence

Immunosenescence represents a fundamental aspect of the progressive functional decline of the immune system associated with aging. It is characterized by a reduction in the generation and activity of immune cells (including B cells, T cells, dendritic cells, and macrophages), an increase in chronic inflammation, and a decline in immune surveillance. These changes significantly elevate the risk of infections, autoimmune diseases, and tumors in the elderly (Condrat et al., 2018; Jaramillo et al., 2020). Recent research has confirmed that epigenetic drift, which includes disordered DNA methylation patterns, abnormal histone modifications, and dysregulated non-coding RNA control, is a crucial molecular mechanism driving immunosenescence. By dynamically regulating the expression of genes involved in immune cell development, differentiation, and function, epigenetic drift plays a central role in the axis of “aging - immune function decline - disease susceptibility,” thereby providing new insights into the mechanisms of immunosenescence and potential intervention strategies (McDonald, 2019; Tabish et al., 2019).

DNA methylation serves as a precise marker of biological age, and its age-related disruption directly impairs immune cell homeostasis. In the hematopoietic system, aging results in decreased expression of TET family enzymes (TET1, TET2, TET3) in hematopoietic stem cells, thereby weakening their proliferation and differentiation capacities. Concurrently, dysfunction arises within the DNMT family; specifically, epigenetic imbalances mediated by DNMT1, a key regulator of B-cell development, lead to disordered hematopoietic lineage output. Further declines in DNMT3A and DNMT3B levels exacerbate the deterioration of HSC regenerative capacity (Hayashi et al., 2020; Sultana and Lee, 2020). Functionally, age-related global hypomethylation of CpG islands can activate pattern recognition receptors, such as Toll-like receptors, which interferes with the immune clearance of apoptotic cells. Research conducted by Sarita Mishra et al. demonstrated that re-methylation of hypomethylated DNA can restore its immunosuppressive properties, indicating that hypomethylated DNA functions as a ‘molecular switch’ for immune dysfunction (McDonald, 2019). Additionally, aberrant methylation of specific genes directly contributes to functional defects in immune cells. For instance, hypermethylation of the IL-7Rα gene promoter inhibits its expression, resulting in decreased proliferative capacity and impaired cytotoxic function of CD8^+^ T cells in the elderly (Sultana and Lee, 2020). Moreover, age-related DNA hypomethylation in dendritic cells enhances their immunogenicity, leading to increased interferon-alpha secretion and exacerbating chronic inflammation and autoimmune responses (McDonald, 2019).

Age-related changes in histone modifications, specifically methylation and acetylation, regulate the transcription of immune-related genes by reshaping chromatin structure, thereby promoting immunosenescence. In aged hematopoietic stem cells, levels of trimethylated histone H3 at arginine 4 (H3R4me3) and arginine 27 (H3R27me3) are significantly increased. This abnormality in histone modifications inhibits the expression of genes related to stem cell differentiation, further compromising the capacity for immune cell production (McDonald, 2019). Conversely, decreased levels of the repressive mark H3K27me3 result in aberrant activation of pro-inflammatory genes, such as tumor necrosis factor-alpha (TNF-α) and IL-6, in immune cells. This exacerbates the chronic inflammatory state while diminishing the efficiency of T-cell responses to pathogens (Bi et al., 2021). Furthermore, the disruption of histone acetylation balance, regulated by HATs and HDACs, also contributes to immunosenescence. Increased HDAC activity with age leads to histone hypoacetylation, which suppresses the expression of immunoregulatory genes, such as IL-10, thereby amplifying inflammatory responses (Neeland et al., 2024).

The age-related reshaping of expression profiles of miRNAs and lncRNAs significantly impacts immune cell function through post-transcriptional regulation. In the case of miRNAs, the expression of miR-21 and miR-146a is notably upregulated in aged immune cells. These miRNAs target immune regulatory genes, such as phosphatase and tensin homolog (PTEN) and Toll-like receptor 4 (TLR4), which inhibits T-cell proliferation and the anti-inflammatory (M2) polarization of macrophages while promoting pro-inflammatory (M1) polarization. This imbalance contributes to the decline in immune function and the persistence of chronic inflammation (Pan et al., 2021; Umbach et al., 2022). Regarding lncRNAs, the expression of lncRNA MALAT1 is downregulated in aged T cells; its overexpression has been shown to enhance T-cell activity and the capacity for anti-tumor immune surveillance by regulating the recruitment of chromatin remodeling complexes. This suggests that lncRNAs may represent potential targets for interventions in immunosenescence (Özyüncü and Erol, 2025).

Drugs that target the reversibility of DNA methylation and histone modifications have demonstrated potential in reversing immunosenescence in preclinical studies. DNMTis can reverse aberrant hypermethylation of genes such as IL-7Rα in T cells, thereby restoring their proliferation and anti-tumor cytotoxic activity (Pilz et al., 2022). HDACis enhance the anti-inflammatory function of macrophages and reduce the secretion of chronic inflammatory factors by increasing histone acetylation levels and activating immunoregulatory gene expression (Neeland et al., 2024). Furthermore, combination strategies that incorporate these drugs with immune checkpoint blockers (ICBs) can significantly improve the efficacy of immunotherapy in elderly cancer patients. Specifically, combining DNMTis with ICBs enhances the anti-tumor response of aged T cells, while HDACis can augment the efficacy of ICBs in elderly patients by reshaping immune cell infiltration within the tumor microenvironment (TME) (Farooqi and Xu, 2024; Helvaci and Yildiz, 2025). The CRISPR-Cas9 epigenome editing technology presents a promising approach for precisely reversing immunosenescence. Targeted editing of abnormal epigenetic sites in immune cells (e.g., the methylation region of the IL-7Rα promoter and genes associated with H3K27me3 modification enzymes) can directly restore immune cell function and delay the immunosenescence process (Taranto et al., 2024). In terms of ncRNA targeting, inhibiting the expression of miR-21 and miR-146a (e.g., via antisense oligonucleotides, ASOs) can alleviate their suppression of immune regulatory genes, thus restoring the anti-inflammatory and immune surveillance functions of T cells (Beddows et al., 2024). Conversely, overexpressing long non-coding RNA (lncRNA) MALAT1 can enhance the activity of aged T cells, providing a novel direction for interventions in immunosenescence (Özyüncü and Erol, 2025).

In summary, epigenetic regulation is a fundamental mechanism driving immunosenescence. Its exploration not only enhances our understanding of the connection between aging and the decline of immune function but also facilitates the development of intervention strategies utilizing epigenetic drugs, gene editing, and targeting of ncRNAs. Future research should further elucidate the immune cell-specific epigenetic regulatory networks, optimize the safety and precision of intervention methods, and provide both theoretical support and a translational basis for improving immune function in the elderly while reducing the risk of immune-related diseases.

### 3.6 Epigenetics of the musculoskeletal system (osteoporosis, sarcopenia)

The aging of the musculoskeletal system presents a significant health challenge for the elderly population, primarily characterized by the high prevalence of OP and sarcopenia, which frequently co-occur as “osteosarcopenia.” The pathological basis of these conditions is closely associated with the age-related imbalance in epigenetic homeostasis (Aurégan et al., 2018; Amano et al., 2023). Epigenetic regulation, including DNA methylation, histone modifications, and ncRNA regulation, dynamically influences the differentiation and function of osteoblasts, osteoclasts, and muscle stem cells (satellite cells), thereby disrupting both bone metabolic balance and muscle regenerative homeostasis. This regulation serves as a crucial molecular link between aging and the functional decline of the musculoskeletal system, providing new insights into disease mechanisms and potential targeted interventions (Davalos and Esteller, 2023; Tung et al., 2023).

During aging, the methylation status of core genes involved in bone metabolism undergoes significant changes. Repe et al. compared healthy individuals with postmenopausal osteoporotic women and found markedly elevated CpG methylation levels in the promoter region of the SOST gene, which encodes the bone formation inhibitor sclerostin, in the latter group. Conversely, Delgado-Calle et al. reported SOST promoter hypomethylation in human bone cells, suggesting that the tissue specificity of SOST methylation may influence bone formation by regulating sclerostin expression (Aurégan et al., 2018). At the level of mesenchymal stem cells (hMSCs), AlvarodelReal et al. conducted epigenomic analyses and identified differences in the methylated regions of genes such as RUNX2, a crucial transcription factor for osteogenic differentiation, and OSX in hMSCs derived from osteoporotic fracture patients compared to healthy individuals. Furthermore, the CpG islands of HOXA (homeobox transcription factors) and RUNX2 in hMSCs from elderly donors exhibited age-related hypermethylation, which directly inhibited their transcriptional activity and diminished the capacity of hMSCs to differentiate into osteoblasts (Tung et al., 2023). Additionally, aging is associated with reduced levels of Tet1/Tet2 (DNA demethylases) in bone marrow stromal cells (BMSCs). Tet1/Tet2 promote osteogenic differentiation by interacting with the Osx promoter enhancer and synergizing with RUNX2 and Dlx5. The age-related hypermethylation of the Tet1/Tet2 genes inhibits RUNX2 expression, leading to osteopenia, a mechanism that has been validated in Tet1/Tet2 knockout mice (Tung et al., 2023).

Imbalances in histone methylation modifications represent a fundamental epigenetic feature of OP. Notably, levels of the repressive mark H3K27me3 increase with age during osteogenesis, with its regulation being dependent on EZH2, a key component of the PRC2 complex. Ren et al. observed an increase in EZH2 expression with age in adult bone marrow aspirates, while Jing et al. further confirmed that EZH2 can inhibit RUNX2 expression by elevating H3K27me3 levels at the RUNX2 transcription site, thereby obstructing osteogenic differentiation (Davalos and Esteller, 2023; Tung et al., 2023). This finding provides a theoretical basis for utilizing H3K27me3 inhibitors, such as EZH2 inhibitors, in the treatment of OP. Concurrently, aberrant activation of HDACs also contributes to bone metabolic disorders. Preclinical studies have demonstrated that HDAC inhibitors, such as SAHA/Vorinostat, promote osteoblast differentiation, inhibit osteoclast activity, and significantly enhance bone microstructure and mechanical properties in osteoporotic model animals (Aurégan et al., 2023; Davalos and Esteller, 2023).

Sarcopenia is characterized by the progressive loss of muscle mass, strength, and function. Its age-related pathology is closely associated with the declined regenerative capacity of muscle stem cells (satellite cells) and the epigenetic silencing of muscle metabolic genes. Comparisons of muscle tissue from young and older individuals have revealed significant differentially methylated CpG sites (dmCpGs) in genes related to cytoskeletal function, axon guidance, muscle contraction, calcium signaling, and the mammalian target of rapamycin (mTOR) pathway (Green et al., 2015). Retrospective cohort studies have confirmed mitochondrial bioenergetic abnormalities and downregulated oxidative phosphorylation gene expression in the skeletal muscle of patients with sarcopenia. These differentially methylated sites are often enriched in EZH2 target genes and regions modified by H3K27me3, suggesting that the synergistic effects of DNA methylation and histone modifications may exacerbate muscle functional decline by suppressing mitochondrial metabolic genes (Zabransky et al., 2022; Amano et al., 2023). Additionally, the disruption of histone acetylation balance contributes to sarcopenia; increased HDAC activity with age leads to histone hypoacetylation, further inhibiting the transcription of muscle-specific genes (e.g., myosin heavy chain (MyHC)) and weakening muscle repair capacity (Davalos and Esteller, 2023).

The age-related reshaping of the miRNA expression profile represents a significant regulatory mechanism in sarcopenia. Specifically, the miR-29 family is upregulated in aged muscle, which accelerates muscle fibrosis and functional degradation by targeting genes involved in collagen synthesis and inhibiting anti-atrophy genes, such as AKT (Green et al., 2015; Gowda et al., 2021).

Current epigenetic interventions targeting the aging of the musculoskeletal system have demonstrated significant potential. HDAC inhibitors, such as SAHA, can concurrently enhance osteoporotic conditions and sarcopenia by promoting osteogenic differentiation in bone tissue and activating muscle regeneration genes in muscle tissue (Aurégan et al., 2023; Davalos and Esteller, 2023). Additionally, DNMT inhibitors can reverse the abnormal hypermethylation of RUNX2 and Tet1/Tet2, thereby restoring the functions of osteogenic and muscle stem cells (Tung et al., 2023). Furthermore, inhibiting miR-29 expression through antisense oligonucleotides (ASOs) has been shown to facilitate muscle regeneration and improve muscle strength in models of sarcopenia (Green et al., 2015).

In conclusion, epigenetic regulation is a fundamental driving factor in the aging of the musculoskeletal system. Research into its mechanisms not only enhances our understanding of the pathological essence of OP and sarcopenia but also fosters the development of targeted drugs and diagnostic technologies. Future efforts should concentrate on the synergistic ‘bone-muscle’ epigenetic network, integrating technological innovations and interdisciplinary approaches to provide precise epigenetic intervention strategies for the musculoskeletal health of the elderly population (Aurégan et al., 2018; Davalos and Esteller, 2023; Tung et al., 2023).

## 4 Targets of aging and epigenetic-targeting anti-aging drugs

Aging is a multifaceted process characterized by the dysregulation of various molecular and cellular mechanisms, with disrupted epigenetic regulation serving as a fundamental driver. Epigenetic alterations, including abnormal DNA methylation, imbalanced histone modifications, and dysfunctional chromatin remodeling, play a direct role in the aging process and the onset of age-related diseases by influencing gene transcriptional activity and determining cell fate. This insight provides a new road for the development of anti-aging drugs that target epigenetic pathways (Imamura et al., 2016; Nagarajan et al., 2019; Peplow, 2024).

### 4.1 DNA methylation modulators: reversing methylation imbalance to restore gene homeostasis

DNA methylation, a fundamental mechanism of epigenetic regulation, exhibits a distinctive disorder in aging characterized by “genome-wide hypomethylation and hypermethylation of specific gene promoters.” This disorder contributes to increased genomic instability and the silencing of genes associated with aging (Teseo et al., 2021). DNA methylation modulators primarily function by inhibiting DNMT activity to reverse abnormal methylation states. Notable examples of these drugs include 5-Azacytidine and Decitabine. In aged mouse models, treatment with 5-Azacytidine has been shown to significantly enhance cognitive function and reduce aging-related inflammatory responses. This effect is attributed to the restoration of the expression of neuroprotective and anti-inflammatory genes that were silenced due to hypermethylation (Yu et al., 2024). Furthermore, these drugs have demonstrated clinical efficacy in treating age-related cancers, such as myelodysplastic syndromes (MDS), as well as metabolic diseases, thereby confirming their broad-spectrum potential in the field of anti-aging research (Zhu et al., 2015). However, their application in non-oncological age-related diseases is limited: for example, they cannot penetrate the blood-brain barrier, making them ineffective for AD; long-term use also increases the risk of genomic instability due to global hypomethylation.

### 4.2 HDAC inhibitors: reshaping chromatin structure to activate repair pathways

HDACs remove acetyl groups from histones, resulting in chromatin condensation and the inhibition of transcription for genes involved in cellular repair and metabolism. Their activity markedly increases with age and is closely linked to neurodegenerative diseases and cardiovascular aging (Maiese, 2018). HDAC inhibitors promote histone acetylation by inhibiting HDAC activity, thereby opening chromatin structure and activating the expression of protective genes. Notable examples of these drugs include Trichostatin A and Vorinostat. Mechanistic studies indicate that HDAC1 deficiency leads to the accumulation of DNA damage and cognitive decline in mouse brains, while HDAC1 activators can enhance cognitive function in aged mice and Alzheimer’s disease model mice by facilitating the recruitment of DNA damage repair enzymes, such as OGG1 (Pao et al., 2020). Additionally, HDAC inhibitors can delay cellular senescence by modulating the expression of mitochondrial respiratory chain genes and reducing the accumulation of oxidative stress products (Tari et al., 2025). Nevertheless, their clinical translation faces challenges: poor tissue specificity (e.g., systemic inhibition of HDACs causes gastrointestinal side effects) and lack of long-term safety data in aging populations.

### 4.3 Histone methylation and chromatin remodeling agents: regulating epigenetic marks to maintain cellular homeostasis

Histone methylation influences chromatin state through the modification of specific lysine and arginine residues, such as H3K27me3 and H3K4me3. An abnormal accumulation of H3K27me3 has been associated with a decline in stem cell functionality and the silencing of aging-related genes (Mortimer et al., 2025). Therapeutic agents targeting this pathway include HMT inhibitors, such as GSK126, which inhibits EZH2 to reduce H3K27me3 levels, and histone demethylase (HDM) inhibitors like JIB-04, which targets the JMJD family to preserve protective methylation marks. These interventions have the potential to restore the balance of histone methylation associated with aging and enhance cell proliferation and differentiation capacities (Doshmangir et al., 2023). Furthermore, dysfunction in chromatin remodeling complexes, such as the SWI/SNF complex, contributes to aging phenotypes, including cardiac fibrosis and neuronal apoptosis. Small molecule modulators of these complexes may improve organ function in aged individuals by restoring chromatin remodeling capabilities, thus representing promising new targets for anti-aging therapies.

### 4.4 Multi-target synergistic therapies: integrating multi-pathway interventions to enhance anti-aging efficacy

Given the synergistic nature of multiple aging mechanisms, single-target drugs often exhibit limited efficacy. Therefore, multi-target synergistic therapy has emerged as a novel trend in epigenetic anti-aging research. This strategy achieves an intervention effect whereby the combined impact of treatments exceeds the sum of their individual effects, denoted as “1 + 1>2,” by jointly regulating multiple epigenetic pathways or by combining with other anti-aging mechanisms. For instance, the combination of HDAC inhibitors and DNA methylation modulators has been shown to significantly enhance metabolic health and cognitive function in aged mice through the synergistic effect of “opening chromatin” and “restoring gene expression” (Duns, 2019). Furthermore, the concurrent application of epigenetic drugs with senolytic agents, which target and eliminate senescent cells, as well as autophagy activators, can effectively suppress both epigenetic dysregulation and the accumulation of senescent cells. In aged mouse models, this combinatorial therapy has been reported to reduce the number of senescent cardiomyocytes, improve exercise endurance, and extend healthspan (Mogilenko et al., 2022; Bujarrabal-Dueso et al., 2025).

In summary, epigenetic drugs that target DNA methylation, histone modifications, and chromatin remodeling have emerged as a pivotal direction for anti-aging interventions. Moreover, multi-target synergistic strategies further enhance their potential applications. Future research should integrate technologies such as single-cell epigenome sequencing and artificial intelligence (AI) in drug design to deepen our understanding of the relationship between epigenetic targets and aging mechanisms. This integration will facilitate the translation of drugs from basic research to clinical applications and provide more precise strategies for delaying aging and treating age-related diseases (Dolan et al., 2025; Poirier, 2025).

## 5 Emerging technologies and challenges

In the field of epigenetic regulation of aging, cutting-edge technologies focusing on precise interventions are progressively overcoming the limitations of traditional methods, thereby offering new avenues for reversing aging-related epigenetic abnormalities. Among these, epigenetic reprogramming technology employs a “partial reprogramming” model to reset aging-associated epigenetic marks while maintaining cell identity, thereby avoiding functional disorders that may arise from full-genome reprogramming. Additionally, CRISPR/dCas9 epigenetic editing technology, which fuses nuclease-inactive Cas9 with epigenetic modification enzymes, facilitates precise regulation of specific targets, such as the p16INK4a gene and telomere-related genes, significantly minimizing non-specific effects (Papathanasiou et al., 2023; Webster and Phillips, 2025). Concurrently, nanocarrier delivery systems, through surface-targeted modifications, accurately deliver drugs such as DNMT inhibitors and HDAC inhibitors to aging-related tissues. Furthermore, single-cell epigenomics technology can elucidate cellular heterogeneity and provide data support for optimizing intervention strategies. Collectively, these technologies enhance the specificity and individual adaptability of interventions (Kalfalah et al., 2015; Gyenis et al., 2023; Kwiatkowska et al., 2023).

However, the clinical translation of these technologies still faces two core challenges. Firstly, the risk of off-target effects is significant: traditional drugs, such as DNMT inhibitors, may induce global epigenetic abnormalities, including hypomethylation of repetitive sequences (e.g., LINE1), activation of proto-oncogenes, or silencing of tumor suppressor genes, which can increase genomic instability and carcinogenic risks (Macedo et al., 2018; Ramini et al., 2022; Schaffer et al., 2023); Even CRISPR/dCas9 editing may lead to epigenetic disorders in adjacent genes due to non-specific binding of guide RNA or sustained activity of modification enzymes (Davalos and Esteller, 2023; Papathanasiou et al., 2023). Secondly, uncontrollable changes in long-term epigenetic memory pose a threat to cell function: improper regulation of partial reprogramming may result in ambiguous cell identity; traditional epigenetic drugs may disrupt stable memories, such as X-chromosome inactivation and genomic imprinting, thereby inducing long-term pathological states, including metabolic disorders and reproductive dysfunction (Waaijer et al., 2016; Kelsey, 2020; Martella et al., 2024).

To address these challenges, future technological optimization should focus on the dual goals of precision and controllability. At the technical level, it is imperative to develop high-specificity CRISPR guide RNA design algorithms and inducible dCas9 systems to achieve spatiotemporally precise regulation of epigenetic modifications. Additionally, optimizing partial reprogramming protocols and integrating dynamic monitoring with single-cell epigenomics are essential to ensure the reversal of aging while preserving cell-specific functions (Papathanasiou et al., 2023; Webster and Phillips, 2025). At the level of personalized intervention, constructing individual aging epigenetic profiles by integrating whole-genome epigenetic sequencing and multi-omics data is crucial. This approach allows for the design of customized protocols targeting specific epigenetic abnormalities associated with different diseases. For instance, individuals exhibiting accelerated DNA methylation age may be prioritized for the targeted delivery of DNMT inhibitors, whereas those with imbalanced histone modifications may benefit from a combination of HDAC inhibitors and epigenetic editing (Davalos and Esteller, 2023; Webster and Phillips, 2025).

From the perspective of clinical translation, future efforts should focus on strengthening the systematic verification of both safety and efficacy. On one hand, large-scale clinical trials are essential to evaluate the long-term safety of cutting-edge technologies, such as assessing the immunogenicity of nanocarriers and the potential impact of CRISPR/dCas9 on germ cells. On the other hand, it is crucial to establish an “epigenetic efficacy evaluation system for aging-related diseases,” utilizing DNA methylation age, organ function parameters, and other indicators as combined endpoints to quantify intervention effects (Davalos and Esteller, 2023; Schaffer et al., 2023). Furthermore, fostering synergy between epigenetic technologies and traditional therapies (e.g., senolytics) to develop a combined intervention model of “epigenetic reset + senescent cell clearance” will ultimately facilitate the transition from basic research to clinical application, offering a new paradigm for the prevention and treatment of aging-related diseases (Davalos and Esteller, 2023; Papathanasiou et al., 2023; Schaffer et al., 2023; Webster and Phillips, 2025).

## 6 Conclusion and perspectives

In recent years, breakthroughs have been achieved in aging epigenetics research centered on three core directions: multi-omics integration, development of novel biomarkers, and ethics in translational medicine (Wu et al., 2021). This review systematically synthesizes the latest advances in this field, which not only deepen the mechanistic understanding of the “epigenetic imbalance–aging–disease” axis but also establish a technical framework bridging basic research and clinical translation. Specifically, the integration of multi-omics technologies and deep learning models enables the prediction of individual-specific epigenetic variations and precise identification of therapeutic targets, even in the absence of samples from hard-to-access tissues (Pan et al., 2022). This lays the foundation for formulating “one-person-one-plan” personalized treatment protocols. Meanwhile, novel biomarkers such as multi-modal aging clocks and liquid biopsies provide powerful tools for non-invasive and accurate monitoring of aging and age-related diseases (Wolf et al., 2023).

Despite the substantial clinical potential of epigenetic interventions (e.g., the successful application of HDAC inhibitors in the treatment of peripheral T-cell lymphoma (PTCL)), their clinical translation still faces three most urgent barriers (Irimia and Piccaluga, 2024). First, tissue-specific drug delivery: targeted delivery systems are urgently needed to improve drug efficacy and reduce off-target effects (Li T. et al., 2024). Second, long-term safety validation: large-scale and long-duration preclinical studies must be conducted to evaluate long-term risks of epigenetic drugs, such as induced genomic instability (Purrucker and Mahlknecht, 2010). Third, development of aging-specific biomarkers: a multi-omics biomarker system integrating DNA methylation, non-coding RNAs, and other omics data needs to be established to accurately assess drug efficacy (Herzog et al., 2024).

Looking ahead, to achieve the successful translation of epigenetic strategies for aging, efforts should focus on the following actionable directions: developing multi-omics biomarkers that can accurately reflect drug effects; designing animal models that better mimic the pathological characteristics of human aging, specifically for aging pharmacology research; and promoting the synergistic application of epigenetic drugs with non-pharmacological interventions such as lifestyle modifications (Liebich et al., 2024). In conclusion, aging epigenetics is not only a core discipline for revealing the essence of aging but also a key force for advancing healthy aging and overcoming age-related diseases. As the field matures and technical bottlenecks are addressed, it will continue to provide solid scientific support for extending human healthspan and improving the quality of life in aging populations.
